# High-throughput microbioreactor provides a capable tool for early stage bioprocess development

**DOI:** 10.1038/s41598-021-81633-6

**Published:** 2021-01-21

**Authors:** Mathias Fink, Monika Cserjan-Puschmann, Daniela Reinisch, Gerald Striedner

**Affiliations:** 1 Christian Doppler Laboratory for Production of Next-Level Biopharmaceuticals in E. Coli, Department of Biotechnology, University of Natural Resources and Life Sciences, Muthgasse 18, 1190 Vienna, Austria; 2 Boehringer Ingelheim RCV GmbH & Co KG, Dr. Boehringer-Gasse 5-11, 1120 Vienna, Austria

**Keywords:** Expression systems, Biotechnology, Protein delivery

## Abstract

Tremendous advancements in cell and protein engineering methodologies and bioinformatics have led to a vast increase in bacterial production clones and recombinant protein variants to be screened and evaluated. Consequently, an urgent need exists for efficient high-throughput (HTP) screening approaches to improve the efficiency in early process development as a basis to speed-up all subsequent steps in the course of process design and engineering. In this study, we selected the BioLector micro-bioreactor (µ-bioreactor) system as an HTP cultivation platform to screen *E. coli* expression clones producing representative protein candidates for biopharmaceutical applications. We evaluated the extent to which generated clones and condition screening results were transferable and comparable to results from fully controlled bioreactor systems operated in fed-batch mode at moderate or high cell densities. Direct comparison of 22 different production clones showed great transferability. We observed the same growth and expression characteristics, and identical clone rankings except one host-Fab-leader combination. This outcome demonstrates the explanatory power of HTP µ-bioreactor data and the suitability of this platform as a screening tool in upstream development of microbial systems. Fast, reliable, and transferable screening data significantly reduce experiments in fully controlled bioreactor systems and accelerate process development at lower cost.

## Introduction

In recent years, both the development of host strains and the identification/design of promising protein candidates have accelerated significantly with steady progress in the fields of molecular biology, genetic engineering, synthetic biology, protein engineering, and bioinformatics. These advances encompass high-throughput (HTP) methodologies, such as HTP proteomics and protein crystallization, microarray technologies, RNA sequencing, large-scale genome sequencing, and gene editing technologies^1–6^.

The construction of combinatorial libraries was strongly supported by growing structural and genome sequence databases^7^. The number of potential protein candidates was boosted by protein engineering approaches utilizing the large number of native protein sequences, designed protein scaffolds, phage displays, and the option to combine functional protein units, such as constant scaffolds with randomized variable regions^8–10^. This is further supported by the use of directed evolution employing machine learning^11^. Another point that broadens the number of variations is the optimization of codon usage to enhance translation efficiency^12^. The number of potential combinations is increased further by the broad range of genetic elements supporting the transcription, translation, and processing of respective proteins of interest^13^.

Antibody fragments as attractive alternatives to full-size antibodies represent a class of proteins with a very broad range of possible combinations. Amongst others the most prominent fragments are the single-chain variable Fragment (scFv) and the antigen binding fragment (Fab). These fragments can be modified using, for example, different variable regions identified via display libraries, by introducing specific sites for chemical conjugation, or by combining different antigen binding sites in divalent F(ab)2 fragments or different linker peptides to connect scFvs. Fragments can also be translocated to the periplasm using different translocation pathways and signal sequences. To improve expression yields, chaperones and trigger factors can be co-expressed, and knocking out proteases can support successful production of antibody fragments^14–16^.

In addition to the high number of possible variants for one protein of interest, there is a vast number of products that potentiate the possible variations to be screened and evaluated. Consequently, there is a large number of host/protein candidates to be evaluated under different production conditions to identify the best possible combination. This challenging task represents one of the main current bottlenecks in the process development chain^17^. In general, bioprocess development is a time-consuming and costly procedure; therefore, reliable and cost-effective HTP cultivation platforms are of utmost importance and central to the process development chain^18,19^.

Shake flask cultivation, the traditional screening method, allows for parallelization, but limited automation and labor intensity severely restrict efficient application in context with HTP approaches. Cultivation in microtiter plates represents an attractive cost-efficient alternative to shaker flasks, as they allow for even higher numbers of parallel cultivations, and the degree of automation via pipetting robotics can be significantly higher^20,21^.

However, in both cases, experiments are performed as batch processes, implying conditions that significantly differ from real production processes with fully controlled cultivation conditions in fed-batch mode. There is no pH control and cells are allowed to grow at maximum rates, which can significantly influence product formation kinetics and cause oxygen limitations. In combination with overflow metabolism caused by high substrate concentrations, these conditions trigger the formation of unwanted byproducts, such as acetate in *Escherichia coli*^22,23^. Acetate formation is one of the most prominent primary processing problems during *E. coli* production processes, as inhibited cell growth and reduced recombinant protein expression result in lower titers and impaired product quality^24^. To increase the transferability of screening results to production processes, screening cultivation conditions should be as close as possible to final production conditions in fed-batch mode^25,26^. Small-scale multi-bioreactor systems designed for mammalian cell culture or microbial systems, are available to run screenings in fed-batch (Ambr15) and intermittent feeding (2mag) mode^27–29^. These devices are already fully functional bioreactors, but they are expensive, their operation quite complex, and nonetheless, the number of experiments limited.

Even though squaring the circle is not possible, advancements in micro titer plate (MTP) cultivation systems significantly enhance the information content of MTP screening experiments. For example, the µ-bioreactor system BioLector provides online data on cell growth, dissolved oxygen, and pH level. This information strongly supports interpretation of HTP experiments during the course of clone and condition screenings^30^. In addition, different techniques allow for carbon-limited growth in MTPs via release of glucose immobilized in a silicone matrix^31^ or via enzymatic cleavage from a dextran base^32,33^. This system can additionally be combined with a liquid handling system. This enables an automated pH control and enzyme addition, resulting in a defined exponential growth and a HTP fed-batch bioprocess, eliminating the limitations in use of enzymatic glucose release caused by a pH shift^34^. MTPs are also available that, with implemented microfluidics, allow media feeds or pH control^35,36^.

In the present study, we evaluated the µ-bioreactor system BioLector operated in an enzymatic substrate release fed-batch mode as a screening cultivation platform with a set of 22 different *E. coli* host/protein/leader combinations. To generate representative benchmark data directly transferable to full-scale process settings, we used either a DASGIP benchtop parallel bioreactor system or a 30 L stirred tank reactor (STR). The main focus was on the extent to which clone rankings based on HTP data match rankings from benchmark experiments conducted in advanced bioreactor systems. The specific recombinant protein concentration was selected as the main ranking.

## Material and methods

### Construction of expression systems

The *E. coli* strains BL21(DE3) (NEB, Ipswich, USA), HMS174(DE3) (Novagen, USA), and RV308 (ATCC # 31608) were used as production hosts. The DE3-derivative of *E. coli* RV308 was prepared using the λDE3-lysogenization kit (Novagen, Germany) according to the manufacturer’s protocol.

Construction of the 16 different genome-integrated (GI) Fab production clones is described in detail in Fink et al.^37^. For expression of FabY and N^pro^MCP1, the pET30a vector (Novagen, Germany; pET System manual, 11th edition) was used. These are described by Schindler et al.^38^. The clones used in this study are listed in Table 1.

**Table 1:** List of all expression clones and abbreviations. <> indicates genomic integration and () indicates plasmid-based systems.

*E. coli* strain	Translocation signal sequence	Model protein	Abbreviation	Reference Experiment
**Part A: Genome integrated expression systems**				
HMS174(DE3)	OmpA^SS^	Fabx	H<oFabx>	1.2 L exponential fed-batch with moderate final cell density of 34 g/L
		BIBH1	H<oBIBH1>	
		BIWA4	H<oBIWA4>	
		FTN2	H<oFTN2>	
	DsbA^SS^	Fabx	H<dFabx>	
		BIBH1	H<dBIBH1>	
		BIWA4	H<dBIWA4>	
		FTN2	H<dFTN2>	
BL21(DE3)	OmpA^SS^	Fabx	B<oFabx>	
		BIBH1	B<oBIBH1>	
		BIWA4	B<oBIWA4>	
		FTN2	B<oFTN2>	
	DsbA^SS^	Fabx	B<dFabx>	
		BIBH1	B<dBIBH1>	
		BIWA4	B<dBIWA4>	
		FTN2	B<dFTN2>	
**Part B: Plasmid-based expression systems**				
HMS174(DE3)	–	N^pro^MCP1	–	20 L exponential fed-batch with final cell density of 115 g/L
	OmpA^SS^	FabY	–	
BL21(DE3)	–	N^pro^MCP1	–	
	OmpA^SS^	FabY	–	
RV308(DE3)	–	N^pro^MCP1	–	
	OmpA^SS^	FabY	–	

### Cultivation

In the course of this study three different bioreactor systems were used, a µ-bioreactor, a fully automated benchtop reactor and a fully automated stirred tank reactor (STR) These showed a final working volume of 800 µL, 1.2 L and 20 L respectively. Table 2 shows a schematic overview of the used reactors, culture time and sampling timepoints.

**Table 2:** Schematic overview of the used reactor systems, culture time (uninduced (ui), induced (i)), and CDM and product sampling timepoints.

Reactor	Culture time (h)	Sampling	
		CDM	Product
µ-bioreactor protocol A	16 (ui) + 9 (i)	Online	25 h
µ-bioreactor protocol B	10 (ui) + 30 (i)	Online	40 h
Benchtop reactor	Feed: 3 (ui) + 16 (i)	Every 2nd hour from induction onwards (3–19)	
STR	Feed: 22 (ui) + 6 (i)	Hourly from induction onwards (22–28)	

#### µ-Bioreactor cultivations

The BioLector system (m2p-labs GmbH, Germany) was used for the µ-bioreactor cultivations. All cultivations were performed at a shaking speed of 1400 rpm and the relative humidity kept at 85%. The biomass was monitored online at 15 min intervals by scattered light measurement at 620 nm, and cell dry mass (CDM) was calculated from a pre-defined calibration curve. Two different protocols were used for cultivation and production.

In the protocol for part A, pre-culture was setup in 48-well Flowerplates B (m2p-labs GmbH) with 800 µL of LB broth (Merck, Germany) at 37 °C. The pre-culture was started with cell material picked directly from frozen working cell banks (WCBs) using pipet tips. For the main culture in 48-well Flowerplates BOH (m2p-labs GmbH), feed-in-time fed-batch medium was prepared according to the m2p-Media development kit (m2p-Labs GmbH) (supplementary data1). This specific type of 48-well plate allows online monitoring of dissolved oxygen (DO) and pH. For inoculation, a defined volume of cell broth from the pre-culture was transferred to achieve a final volume of 800 µL with an initial OD_600_ of 0.3; the cultivation temperature was 30 °C. Induction was performed with a final concentration of isopropyl-β-D-thiogalactopyranoside (IPTG) (Carl Roth, Germany) of 0.5 mM 16 h after inoculation. The production phase was specified to be 9 h, and endpoint samples were drawn for product analysis^37^.

In the protocol for part B, the inoculum for the main culture was directly prepared from WCBs as described by Toeroek et al.^25^. Inoculation was performed to reach an initial OD_600_ of 0.5 in a total volume of 800 µL. For the main culture in BOH plates the premixed FIT-medium (m2p-Labs GmbH) diluted to 67%, was used and the cultivation temperature was set to 30 °C. The induction time point was 10 h after inoculation with a final concentration of 1 mM IPTG.

#### Benchtop fed-batch cultivation

For the benchtop fed-batch cultivations, we used a DASGIP parallel bioreactor system (Eppendorf AG, Germany) enabling four parallel cultivations. The total vessel volume was 2.1 L with a maximum working volume of 1.8 L The bioreactors were equipped with a pH probe (Hamilton Bonaduz AG, Switzerland), an optical DO probe (Hamilton Bonaduz AG), and a DASGIP GA4X-module (Eppendorf AG) for online off-gas monitoring. For media preparation, all chemicals were purchased from Carl Roth GmbH (Germany) if not stated otherwise.

Pre-culture was used for inoculation of the bioreactors. For pre-culture, the cells were incubated in 50 mL of a semi synthetic medium (SSM) in 500 mL baffled glass flasks at 37 °C with shaking at 180 rpm. The SSM was prepared from 3.00 g/L KH_2_PO_4_ and 4.58 g/L K_2_HPO_4_, enabling a calculated CDM of 3 g/L. The following components were added in amounts per gram of CDM: 0.1 g tryptone, 0.05 g yeast extract (Merck, Germany), 0.25 g C_6_H_5_Na_3_O_7_·2H_2_O, 0.1 g MgSO_4_·7H_2_O, 0.01 g CaCl_2_·2H_2_O, 50 µL trace element solution, 0.45 g (NH_4_)_2_SO_4_, 0.37 g NH_4_Cl, and 3.30 g C_6_H_12_O_6_·H_2_O.

The HMS174(DE3) pre-cultures were inoculated with 1250 µL thawed WCB and incubated for approximately 8 h to reach a final OD_600_ of 2.8–3.8. For BL21(DE3), 50 µL of thawed WCB was used for inoculation and the cells incubated for approximately 7 h. Depending on the final OD_600_, a defined volume of pre-culture was transferred to the bioreactors to reach an initial OD_ini_ of approximately 0.04.

The fermentation process was designed for a final amount of 40 g CDM of which 6 g were obtained in a batch volume of 600 mL and 34 g during feed phase via the addition of another 600 mL of feed medium. The amount of glucose for the specific medium was calculated based on a yield coefficient (Y_x/s_) of 0.3 g/g and added as C_6_H_12_O_6_·H_2_O. The following components were added to the batch medium according to the final biomass of the process due to solubility issues in the feed medium: 0.094 g KH_2_PO_4_, 0.032 g 85% H_3_PO_4_, 0.041 g C_6_H_5_Na_3_O_7_·2H_2_O, and 0.045 g (NH_4_)_2_SO_4_. According to the grams of CDM formed during batch phase, the following components were added: 0.15 g yeast extract (Merck), 0.046 g MgCl_2_·6H_2_O, 0.202 g CaCl_2_·2H_2_O, and 50 µL trace element solution. The feed medium was composed of the following components according to the grams of CDM formed during the feed phase: 0.046 g MgCl_2_·6H_2_O, 0.202 g CaCl_2_·2H_2_O, and 50 µL trace element solution.

During batch phase, the temperature was maintained at 37 °C ± 0.2. The feed was started at the end of the batch phase, and simultaneously the cultivation temperature was set to 30 °C ± 0.2. During the feed phase, an exponential carbon-limited substrate feed was provided to maintain a constant growth rate of 0.1 h^-1^. The weight loss-controlled substrate feed was conducted by increasing the pumping rate according to the exponential growth formula $x={x}_{0}{e}^{\mu t}$. During the whole process, the pH was maintained at 7.0 ± 0.2 by the addition of 12.5% ammonia solution. The dissolved oxygen saturation was stabilized above a level of 30% by cascade control of the stirrer speed, aeration rate, and gassing composition. Foaming was suppressed by the addition of 750 µL of PPG 2000 (BASF, Germany) to the batch medium and by the automatic addition of 1:10 diluted PPG 2000 controlled by a conductivity operated level sensor. Protein expression was induced by IPTG after approximately 0.4 doublings during the feed phase for approximately 2.3 more doublings. The induction level was chosen to yield a constant concentration of 2 µmol IPTG/g of theoretical CDM. This means an appropriate amount of IPTG was added sterile to the bioreactor at induction time point and to the remaining feed medium, i.e. 3.86 and 15.20 mg dissolved in RO-H_2_O respectively.

#### High cell density cultivations

High cell density (HCD) cultivations were performed in a fully automated 30 L STR (Bioengineering, Switzerland) with a working volume of 23 L. A batch volume of 10 L was used. The pH was set to 7.0 ± 0.5 and maintained by the addition of 25% ammonia solution. During the whole fermentation, the temperature was maintained at 30  ± 0.5 °C except for N^pro^MCP1 fermentations, which were performed at 37  ± 0.5 °C. The DO level was set to 30% by adjusting stirrer speed, aeration rate, and overpressure at the head space, which was set to a maximum of 1.0 bar. Foam control was performed by the addition of 0.5 mL/L of PPG 2000 (Sigma Aldrich, USA) to the batch medium and pulsed addition during the fed-batch phase. For pre-culture, 300 mL of LB media was inoculated with 1 mL of thawed WCB and incubated at 37 °C and 200 rpm up to an OD_600_ of 3.5. For inoculation of the STR, a total of approximately 15 mL of pre-culture was used. After the batch phase, an exponential substrate feed was started, maintaining a constant growth rate of 0.17 h^-1^ for a duration of 11 h. The substrate feed was performed by increasing the pumping rate according to the exponential growth formula $x={x}_{0}{e}^{\mu t}$ using scale feedback control. Afterwards, the exponential feed phase regulation was changed to a linear feed phase of 17 h, providing a constant rate of 4.9 g glucose per minute, resulting in a decreasing growth rate from 0.17 to 0.04 h^−1^. Media were composed to gain 80 g CDM during the batch phase and 1940 g CDM during the feed phase. The feed medium was prepared gravimetrically with a final weight of 10.2 kg. Details for media preparation are given in Toeroek et al.^25^.

#### Off-line analysis

The benchtop reactor for off-line analysis (OD_600_, CDM, product) was sampled during the production phase at a frequency of 2 h. The samples after 8 and 16 h of production were then used for direct comparison with the µ-bioreactor results. The OD_600_ was measured using an Ultrospec 500 pro Spectrophotometer (Amersham Biosciences, UK), diluting the samples with phosphate-buffered saline to achieve the linear range of measurement. For the determination of CDM, 1 mL of cell suspension was transferred to pre-weighed 2.0 mL reaction tubes and centrifuged for 10 min at 16,100 rcf and 4 °C with an Eppendorf 5415 R centrifuge. The supernatant was transferred to another reaction tube and stored at -20 °C for further analysis. As a washing step, the cell pellet was resuspended in 1.8 mL RO-H_2_O, centrifuged, and the supernatant discarded. Afterwards, the pellet was resuspended in 1.8 mL RO-H_2_O and finally dried at 105 °C for 24 h. Before re-weighing the reaction tubes, they were cooled to room temperature in a desiccator.

For intracellular product analysis, the sampling volume of the cell suspension corresponding to 1 mg CDM was estimated via direct measurement of the OD_600_. The calculated amount was transferred to 1.5 mL reaction tubes and centrifuged at 16,100 rcf and 4 °C for 10 min. The supernatant was discarded, and the cell pellets stored at − 20 °C.

Fab quantification was performed using a sandwich enzyme-linked immunosorbent assay (ELISA). This specific ELISA only detects correctly assembled Fab by capturing it with an antibody that binds the light chain and detecting it with an antibody binding specifically to the hinge region of the heavy chain. For sample preparation chemical cell lysis was performed to gain the soluble intracellular Fab fraction. These soluble fractions then were diluted to be in the range of 0.78 ng/mL to 100 ng/mL, which was the standard curve calculated using purified human Fab/κ (Bethyl P80-115; USA). Measurements were executed at absorbances at 492 nm and 620 nm. The detailed protocol was performed according to former work in our group^37^.

## Results and discussion

### Transferability of µ-bioreactor Fab clone screenings to the benchtop bioreactor

In this first part of the study, the Fab production clone characterizations in fed-batch-like HTP µ-bioreactor cultivations were evaluated with respect to their transferability to fed-batch data from benchtop bioreactor cultivations. For this purpose, we selected a set of 16 different GI Fab production clones (Table 1A). The 1.2 L benchtop bioreactor cultivations were operated in fed-batch mode with an exponential feed profile, providing a growth rate of 0.1 h^−1^ and a final cell density set to 30 g/L. This reference process scheme represents the final development stage that is directly transferable to pilot and production scales.

#### µ-Bioreactor cultivations of different Fab expression systems

**Figure 1:**
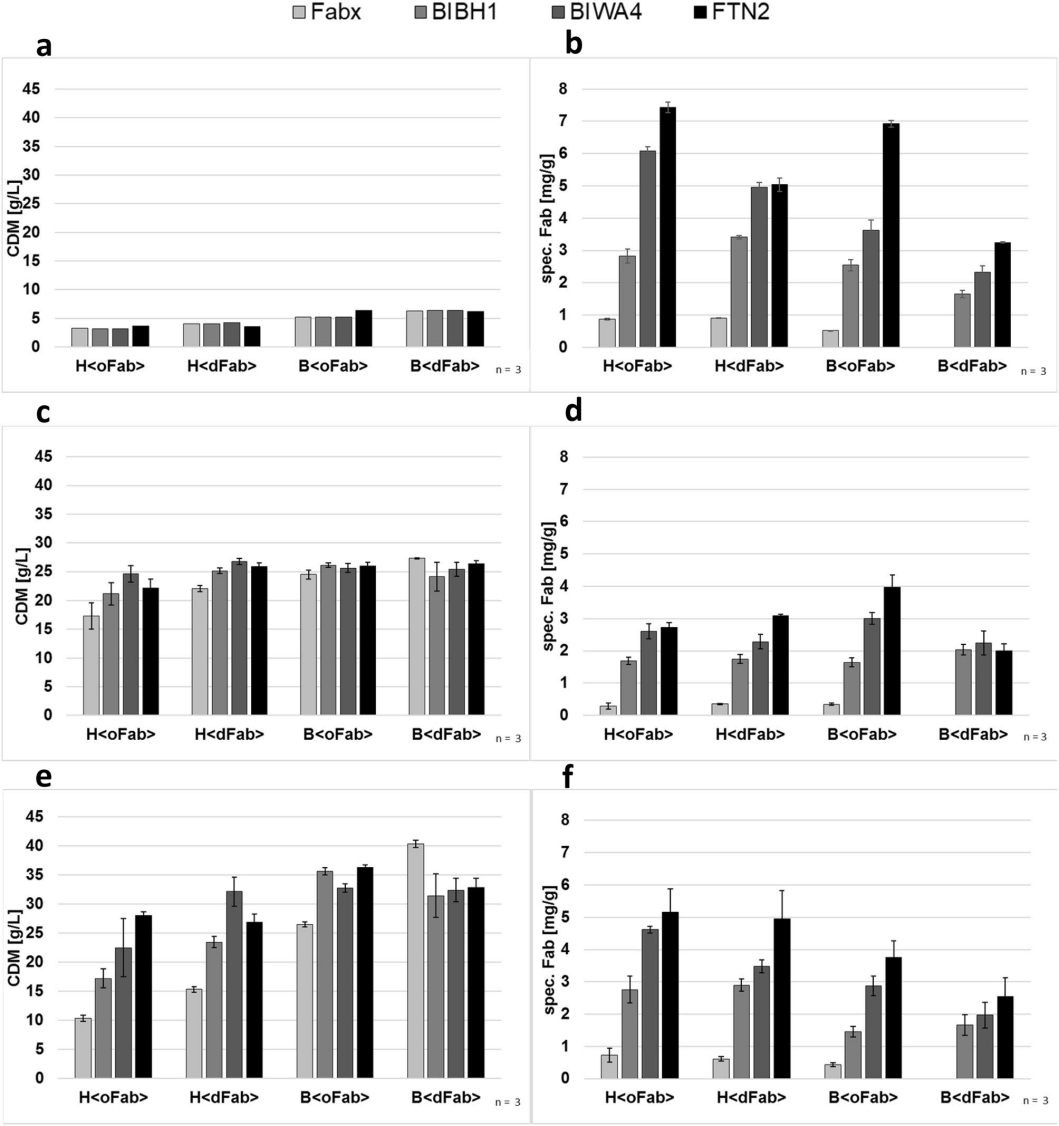
Final CDM for all host-leader-Fab combinations in the µ-bioreactor **(a)** and CDM in benchtop cultivations after 8 h production phase **(c)** and at the end of fermentation **(e)**. Soluble Fab yields for all expression systems in µ-bioreactor cultivations **(b)** and benchtop cultivations after 8 h production phase **(d)** and at the end of fermentation **(f)**. Data are presented as mean ± SD. n indicates biological replicates.

Cell growth, pH, and oxygen saturation were monitored online throughout the whole µ-bioreactor cultivations. No oxygen limitations were observed and pH values maintained within a range of 7 to 6.7. In contrast to shake flask experiments, the fed-batch-like cultivation mode resulted in carbon-limited growth with low and almost constant growth rates during the Fab production phase. Independent from recombinant gene expression, the main variation in final cell density was caused by the host cell line, with higher cell densities for BL21(DE3) clones. As illustrated in Fig. 1a, all B<dFab> clones and the B<oFTN2> clone had biomass yields around 6 g/L. The remaining three B<oFab> clones yielded final biomass concentrations of approximately 5 g/L. The production host HMS174(DE3) had lower final cell concentrations of approximately 4 g/L for H<dFab> clones and 3 g/L for H<oFab> clones (Fig. 1a). These results indicate a significant negative influence of the ompA^SS^ leader on the cell growth of both host strains. However, a closer look at Fab expression data revealed explicitly higher Fab yields for almost all clones with the post-translational ompA^SS^ translocation signal. Fab expression was evaluated by the amount of correctly processed and soluble Fab in the periplasm which was determined by ELISA. The different Fab and signal sequence combinations resulted in significantly variable expression levels in both host strains (Fig. 1b). The worst performing clone was B<dFabx> , as it had no detectable Fab expression. For all other production clones, we observed specific Fab concentrations ranging from 0.01 to 7.4 mg/g CDM. With respect to the expression levels of individual Fabs, all host-leader combinations end up with the same ranking, with highest expression levels for FTN2, followed by BIWA4, BIBH1, and Fabx. With respect to specific Fab yields, HMS174(DE3) clones always outcompete respective BL21(DE3) variants.

#### Benchtop bioreactor cultivations of different Fab expression systems

**Figure 2:**
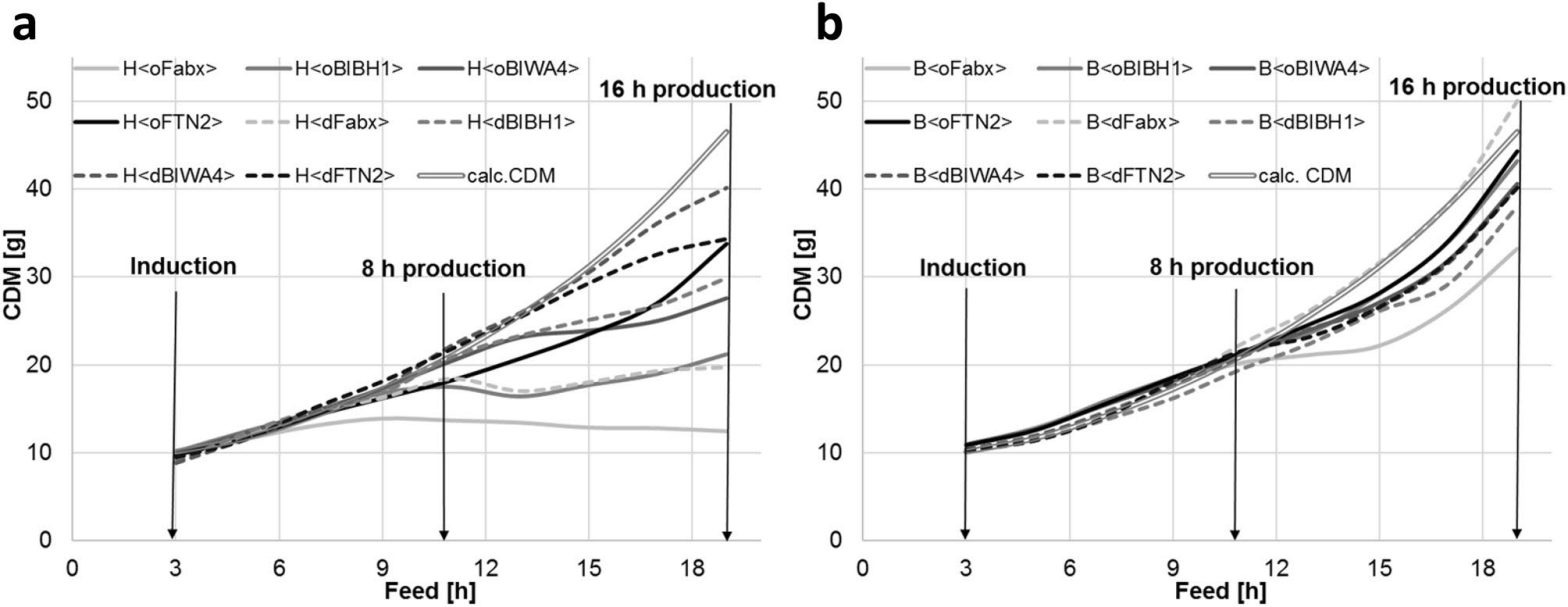
Growth kinetics for the benchtop fed-batch cultivation during production phase of the expression clones. **(a)** HMS174(DE3) and **(b)** BL21(DE3) expressing the four Fabs with solid lines for OmpA^ss^ and dashed lines for DsbA^ss^. A double line is used for the theoretical CDM. The induction time point is indicated by the arrow at 3 h of feed phase. The arrows at 8 and 16 h past induction indicate the data obtained at these timepoints shown in detail in Fig. 1.

Compared to µ-bioreactor experiments, the experimental design for benchtop bioreactor cultivations resulted in a higher growth rate of 0.1 h^−1^ in the feed and production phases. The production phase with 16 h was significantly longer, and the difference was even more pronounced with respect to cell doublings. Results were compared after 8 and 16 h of production; Fig. 2 presents the growth kinetics during the production phase for the HMS174(DE3) (Fig. 2a) and BL21(DE3) (Fig. 2b) clones. First, we examined the results after 8 h of production, which is approximately the same period as in the 9-h µ-bioreactor setting (Fig. 1c,d). In this setting, all BL21(DE3) clones and all H<dFab> clones reached biomass concentrations of 25 ± 1.5 g/L. We observed lower biomass yields only for H<oFab> clones, with significant variation from 17 ± 1.5 g/L for H<oFabx> up to 25 ± 1.5 g/L for H<oBIWA4> . With respect to Fab yields, specific concentrations were approximately 50% lower than in the titer plate experiments, which were driven mainly by higher growth rates, which were already found to be counterproductive in that context.

Next, we examined the results after 16 h of production at the end of the cultivation phase to evaluate the impact of an elongated production phase on product yields. We observed more pronounced adverse effects of Fab production on cell growth, as the final biomass concentrations of the different clones exhibited greater variety. The final CDM yields for BL21(DE3) varied from 26.5 to 40.4 g/L, whereas HMS174(DE3) only reached CDM concentrations of 10.4 to 32.1 g/L (Fig. 1e). Additionally, from the growth kinetics (Fig. 2) it gets apparent that the impact on growth starts approximately 9 h after induction for almost all clones. This impact is more pronounced for HMS174(DE3) clones. As shown in a previous study^37^, the expression of Fabx had the worst effect on growth in both strains. We found no detectable Fab expression in the B<dFabx> strain, which is also the strain with the highest CDM. Expression clones with the dsbA^ss^ leader reached higher CDMs, with the exception of H<FTN2>.

#### Direct comparison and significance of µ-bioreactor Fab clone screening

Comparing both cultivation scales, the main finding of the µ-bioreactor experiments, strong dependency of expression on the individual Fab molecule, was confirmed in the benchtop bioreactor experiments. They showed exactly the same ranking of specific Fab titers after 16 h (Fig. 1f) as expression for each host/leader combination after 8 h (Fig. 1d); it was also the same except for B<dFab> , where FTN2 was expressed at lower levels than BIBH1 and BIWA4. This resulted in a specific Fab concentration range of 0.0 to 5.2 mg/g CDM after 16 h and 0.0 to 4.0 mg/g CDM after 8 h. In regards to the overall ranking of the different hosts, the transferability depends on the expression period. After 16 h (Fig. 1f) of expression, the ranking of host leader combinations was exactly the same as in the µ-bioreactor, starting with H<oFab> and followed by H<dFab> , B<oFab> , and B<dFab> . Thus, there is a distinct trend favoring HMS174(DE3) as expression host and ompA^SS^ as leader sequence. Considering the final CDM after this period (Fig. 1e), the results from the µ-bioreactor (Fig. 1a) were replicated in the benchtop bioreactor. In both systems, almost all BL21(DE3) clones achieved higher CDM than the respective HMS174(DE3) counterpart. Although this was less pronounced after 8 h (Fig. 1c), the tendency was already indicated. Even though H<oBIBH1> , for example, reached the same titer in both reactor systems, the maximum titer in the benchtop reactor was approximately 20% lower on average than in the µ-bioreactor. This may be caused by differences in growth rate profiles during the expression phase, which is constant at 0.1 h^−1^ and approximately 3-times higher in the benchtop bioreactors compared to the steadily decreasing growth rate in the µ-scale system. This assumption was already confirmed with experiments at a growth rate of 0.05 h^−1^ (data not shown).

### Transferability of µ-bioreactor to HCD productions

**Figure 3:**
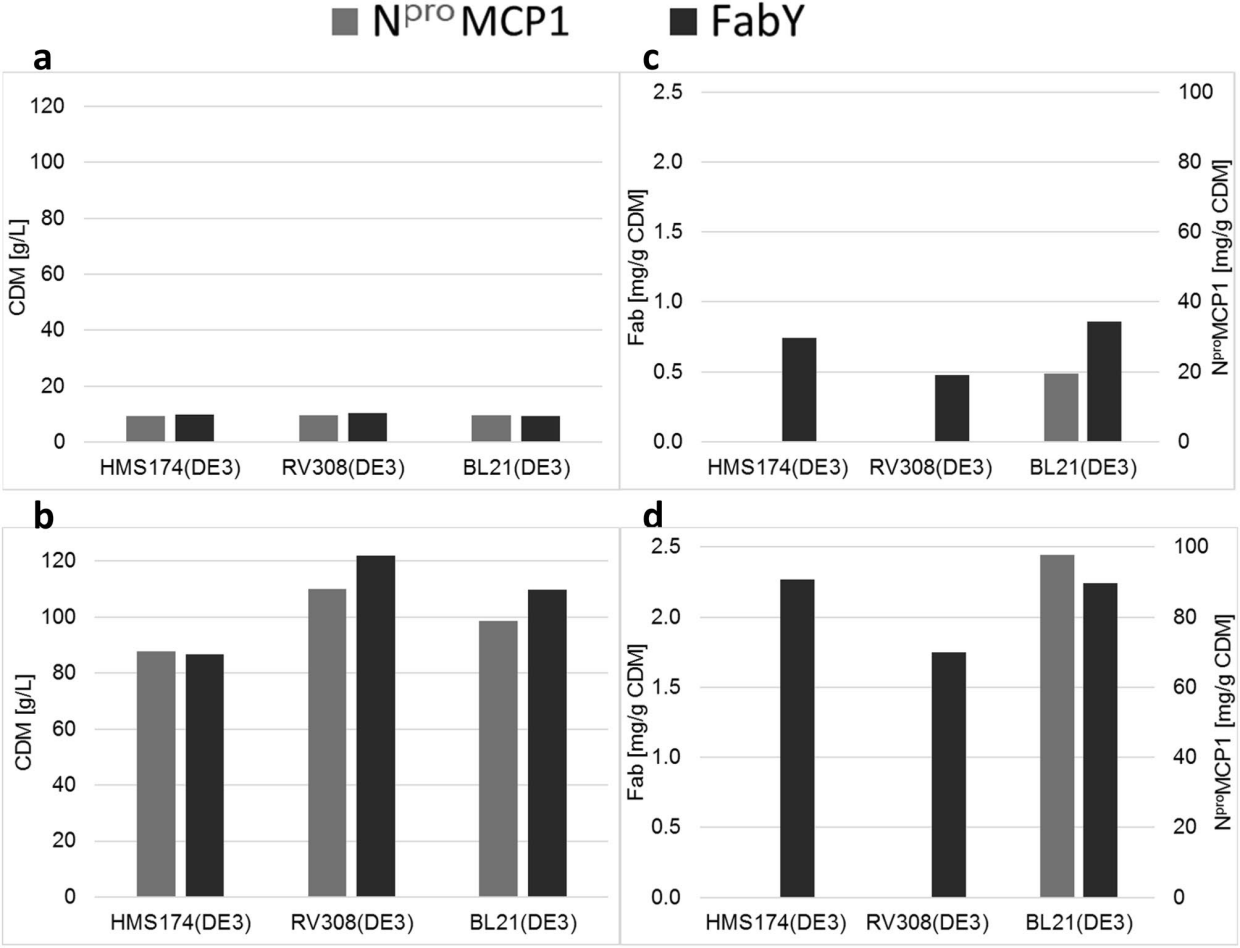
Final CDM (g/L) of all six plasmid-based production clones from µ-bioreactor **(a)** and HCD cultivations **(b)** with the corresponding total specific product yields (**c,d**, respectively). Fab product yields are indicated on the primary ordinate and N^pro^MCP1 on the secondary ordinate.

The main goal of these experiments was to evaluate the transferability and reliability of HTP µ-bioreactor clone screening results to HCD production. In this part of the study, we added *E. coli* RV308(DE3) as a third host strain and selected FabY as a periplasmic protein and N^pro^MCP1 as an insoluble cytosolic fusion protein, both encoded by plasmids. Again, we defined the specific product concentration as the main transferability ranking criterion (Fig. 3).

#### Fab clone characteristics

With respect to cell growth for final CDM, we observed only slight differences between the three strains in the µ-bioreactor experiments (Fig. 3a), ranging from 10.3 g/L CDM for RV308(DE3) to 9.3 g/L for BL21(DE3). Detailed results for growth kinetics were published previously^25^. In the HCD process (Fig. 3b), the differences were much more pronounced, with the highest final biomass concentration of 122 g/L for RV308(DE3) and only 86.7 g/L for HMS174(DE3). We found a significant influence of HCD conditions on the growth performance of the three strains, and higher FabY expression levels can amplify these effects.

µ-Bioreactor cultivations showed that plasmid-based Fab production clones of BL21(DE3) and HMS174(DE3) resulted in similar specific Fab yields of 0.86 and 0.74 mg/g CDM, respectively. The RV308(DE3) strain had a significantly lower yield of only 0.48 mg/g CDM (Fig. 3c). Direct comparison with the results from HCD cultivations (Fig. 3d) confirmed the µ-bioreactor ranking with higher, and again similar, specific Fab concentrations of 2.24 mg/g CDM for BL21(DE3) and 2.27 mg/g CDM for HMS174(DE3), and a lower Fab level of 1.75 mg/g CDM for RV308(DE3). In this approach, growth rates were significantly lower than above, which confirms the assumption that high growth rates may cause the lower Fab titers in benchtop cultivations.

Although the observed differences between the three Fab production clones were not that distinct in the µ-bioreactor experiments, the same trends as in the HCD-cultivation were found and consequently facilitated an acceptable clone assessment.

#### N^pro^MCP1 production clone characteristics

The second model protein used during this evaluation was N^pro^MCP1 expressed in the cytosol. In µ-bioreactor experiments, we observed that HMS174(DE3) and the RV308(DE3) system did not express any product, even though accurate cloning was confirmed via sequencing and no plasmid loss identified, though BL21(DE3) reached a specific product concentration of approximately 19.6 mg/g CDM. This unexpected result was confirmed in HCD cultivation, with no expression by HMS174(DE3) and RV308(DE3) and a rather high specific concentration of approximately 98 mg/g CDM for BL21(DE3). Regarding cell growth, the results from the µ-bioreactor system revealed no significant variation between the three strains, ranging from 9.7 g/L for BL21(DE3) to 9.3 g/L for HMS174(DE3). In HCD cultivation, HMS174(DE3) showed the worst growth performance with a final concentration of only 87.7 g/L, though no product formation was observed.

In summary, we could show that the µ-bioreactor results provide a solid basis for selecting clones for HCD cultivation. In addition, we observed a severe growth problem for HMS174(DE3) under HCD conditions independent from recombinant protein expression.

## Conclusion

The results showed that HTP µ-bioreactor cultivations are capable of identifying potentially promising production clones for further experiments in the course of bioprocess development. The first main finding was that the expression level of the four different Fabs ranked as FTN2 > BIWA4 > BIBH1 > Fabx and was the same independent of the leader sequence and host strain. In addition, HMS174(DE3) and ompA^SS^ were superior to their respective counterparts, BL21(DE3) and dsbA^SS^. These ranking results from the µ-bioreactor were reproduced in the benchtop bioreactors, though lower specific product yields were obtained in this setting. In context with process optimization, the results also revealed a strong correlation between the growth rate and Fab expression level independent from strain, signal sequence, and Fab candidate. Second, with plasmid-based expression systems, transferability of µ-bioreactor results to HCD cultivations was very good even though the rankings were not always identical. However, the limited growth performance of HMS174(DE3) in HCD culture was one of the main reasons for the slight ranking differences between the two scales.

With regard to the robustness of the approach, it must be clearly stated that the conditions in the microbioreactor can never exactly reproduce those of the larger scale. Consequently, a successful application of the microbioreactor system requires a precise analysis of the conditions in both scales and, based on this, the closest possible approximation of the settings in the microscale to the conditions in the production process.

In the course of this evaluation we also identified some limitations of the µ-bioreactor system and its use as a HTP screening platform, especially in a context with control options. Active control of pH and growth rate, which both can have a significant influence on recombinant gene expression levels, is not possible. This problem is indirectly solved by using FIT medium with a strong buffer capacity in combination with enzyme-based glucose release. However, this solution results in higher osmotic pressure and differences in media composition compared to stirred tank bioreactor systems, which can also influence product quantity and quality. Compared to the next generation BioLector Pro or more complex multi bioreactor systems, there is a clear trade-off in terms of throughput, cost, and explanatory power of the data. With awareness of the given limitations of the µ-bioreactor platform, it can efficiently be used as a powerful, time-saving, and cost-effective screening tool in early-stage process development, delivering reliable data that can serve as the basis for making decisions about the design of subsequent development steps.

## Supplementary Information

Supplementary Information.

## Abbreviations

CDM: Cell dry mass
DO: Dissolved oxygen
Fab: Fragment antigen binding
GI: Genome integrated
HCD: High cell density
HTP: High-throughput
µ-bioreactor: Micro-bioreactor
MTP: Micro titer plate
scFv: Single-chain variable fragment
SS: Signal sequence
STR: Stirred tank reactor
WCB: Working cell bank

## References

[1] Hui R, Edwards A (2003). High-throughput protein crystallization. J. Struct. Biol..

[2] Rogers YH, Venter J (2005). C. Genomics: Massively parallel sequencing. Nature.

[3] Lee SY, Mattanovich D, Villaverde A (2012). Systems metabolic engineering, industrial biotechnology and microbial cell factories. Microb. Cell Factories..

[4] Zheng X, Xing XH, Zhang C (2017). Targeted mutagenesis: A sniper-like diversity generator in microbial engineering. Synth. Syst. Biotechnol..

[5] Shanmugam S, Ngo HH, Wu YR (2020). Advanced CRISPR/Cas-based genome editing tools for microbial biofuels production: A review. Renewable Energy.

[6] Berlec A, Strukelj B (2013). Current state and recent advances in biopharmaceutical production in *Escherichia coli*, yeasts and mammalian cells. J. Ind. Microbiol. Biotechnol..

[7] Hosse RJ, Rothe A, Power BE (2006). A new generation of protein display scaffolds for molecular recognition. Protein Sci..

[8] Bradbury ARM, Marks JD (2004). Antibodies from phage antibody libraries. J. Immunol. Methods.

[9] Binz HK, Amstutz P, Plückthun A (2005). Engineering novel binding proteins from nonimmunoglobulin domains. Nat. Biotechnol..

[10] Binz HK, Plückthun A (2005). Engineered proteins as specific binding reagents. Curr. Opin. Biotechnol..

[11] Yang KK, Wu Z, Arnold FH (2019). Machine-learning-guided directed evolution for protein engineering. Nat. Methods.

[12] Klumpp S, Dong J, Hwa T (2012). On ribosome load, codon bias and protein abundance. PLoS ONE.

[13] Gileadi, O. in *Methods in Molecular Biology* Vol. 1586 3–10 (2017). 10.1007/978-1-4939-6887-9_1 28470595

[14] Frenzel A, Hust M, Schirrmann T (2013). Expression of recombinant antibodies. Front. Immunol..

[15] Gebauer M, Skerra A (2009). Engineered protein Scaffolds as next-generation antibody therapeutics. Curr. Opin. Chem. Biol..

[16] Gupta SK, Shukla P (2017). Microbial platform technology for recombinant antibody fragment production: A review. Crit. Rev. Microbiol..

[17] Bareither R, Pollard D (2011). A review of advanced small-scale parallel bioreactor technology for accelerated process development: Current state and future need. Biotechnol. Prog..

[18] Hemmerich J, Noack S, Wiechert W, Oldiges M (2018). Microbioreactor systems for accelerated bioprocess development. Biotechnol. J..

[19] Panula-Perälä J (2008). Enzyme controlled glucose auto-delivery for high cell density cultivations in microplates and shake flasks. Microb. Cell Factories..

[20] Prado RC, Borges ER (2018). Microbioreactors as engineering tools for bioprocess development. Braz. J. Chem. Eng..

[21] Velez-Suberbie ML (2018). High throughput automated microbial bioreactor system used for clone selection and rapid scale-down process optimization. Biotechnol. Prog..

[22] Büchs J (2001). Introduction to advantages and problems of shaken cultures. Biochem. Eng. J..

[23] Jeude M (2006). Fed-batch mode in shake flasks by slow-release technique. Biotechnol. Bioeng..

[24] Faulkner E (2006). Use of fed-batch cultivation for achieving high cell densities for the pilot-scale production of a recombinant protein (phenylalanine dehydrogenase) in *Escherichia coli*. Biotechnol. Prog..

[25] Toeroek C, Cserjan-Puschmann M, Bayer K, Striedner G (2015). Fed-batch like cultivation in a micro-bioreactor: Screening conditions relevant for *Escherichia coli* based production processes. SpringerPlus..

[26] Keil T, Landenberger M, Dittrich B, Selzer S, Büchs J (2019). Precultures grown under fed-batch conditions increase the reliability and reproducibility of high-throughput screening results. Biotechnol. J..

[27] Warr, S. R. C. in *Methods in Molecular Biology* Vol. 2095 43–67 (2020). 10.1007/978-1-0716-0191-4_4 31858462

[28] Kreye S (2019). A novel scale-down mimic of perfusion cell culture using sedimentation in an automated microbioreactor (SAM). Biotechnol. Prog..

[29] Janzen NH (2019). Implementation of a fully automated microbial cultivation platform for strain and process screening. Biotechnol. J..

[30] Lladó Maldonado S (2019). A fully online sensor-equipped, disposable multiphase microbioreactor as a screening platform for biotechnological applications. Biotechnol. Bioeng..

[31] Keil T, Dittrich B, Lattermann C, Habicher T, Büchs J (2019). Polymer-based controlled-release fed-batch microtiter plate—Diminishing the gap between early process development and production conditions. J. Biol. Eng..

[32] Török, C. *Screening of recombinant Escherichia coli host strains in a micro-bioreactor applying a fed-batch cultivation protocol. A way to accelerate process development?* (University of Natural Resources and Life Sciences, Vienna, 2014).

[33] Krause M (2010). A novel fed-batch based cultivation method provides high cell-density and improves yield of soluble recombinant proteins in shaken cultures. Microb. Cell Factor..

[34] Jansen R (2019). FeedER: A feedback-regulated enzyme-based slow-release system for fed-batch cultivation in microtiter plates. Bioprocess Biosyst. Eng..

[35] Blesken C, Olfers T, Grimm A, Frische N (2016). The microfluidic bioreactor for a new era of bioprocess development. Eng. Life Sci..

[36] Morschett H (2020). Parallelized microscale fed-batch cultivation in online-monitored microtiter plates: Implications of media composition and feed strategies for process design and performance. J. Ind. Microbiol. Biotechnol..

[37] Fink M (2019). Microbioreactor cultivations of Fab-producing *Escherichia coli* reveal genome-integrated systems as suitable for prospective studies on direct Fab expression effects. Biotechnol. J..

[38] Schindler S (2016). Npro fusion technology: On-column complementation to improve efficiency in biopharmaceutical production. Protein Expr. Purif..

