# Supplementary material for: High-throughput microbioreactor provides a capable tool for early stage bioprocess development
Source: Sci Rep. 2021 Jan 21;11:2056. doi: 10.1038/s41598-021-81633-6 (PMC7819997; doi:10.1038/s41598-021-81633-6)
Supplement: Supplementary file 1 — Supplementary Information. [file 41598_2021_81633_MOESM1_ESM.pdf]

Supplementary data1

High-throughput microbioreactor provides a capable tool for early stage bioprocess development

Mathias Fink[1], Monika Cserjan-Puschmann[1], Daniela Reinisch [2], Gerald Striedner[1]

[1] Christian Doppler Laboratory for Production of Next-Level Biopharmaceuticals in *E. coli*, Department of Biotechnology University of Natural Resources and Life Sciences Muthgasse 18 A - 1190, Vienna, Austria

[2] Boehringer Ingelheim RCV GmbH & Co KG Dr. Boehringer-Gasse 5-11 A-1120, Vienna, Austria

Correspondence: Monika Cserjan-Puschmann

E-Mail: monika.cserjan@boku.ac.at

µ-bioreactor medium for *E. coli* cultivation

The media development kit recipe is designed for 100 mL and can be adjusted as required. For complex composition 0.5 % w/v wheat peptone can be added to the Salt mix as a starting point for optimization before adjustment in further development.

The finalized medium and the stock solutions, except the polysaccharide solution ought to be stored refrigerated at 4 °C, the polysaccharide solution at room temperature. The durability of all media solutions is minimum 3 months. If crystalline precipitation is observed the solutions have to be discarded. If polysaccharide solution shows a precipitate it has to be warmed to 30 °C and mixed thoroughly to get a clear solution again.

Salt mix

Components	Weight [g]
MOPS	4.19
$(NH_4)_2SO_4$	1.00
K_2HPO_4	0.30
$Na_3Citrate \cdot 2\ H_2O$	0.30
Na_2SO_4	0.20
NH_4Cl	0.10
**For complex composition:** Wheat peptone	0.50
Deionized water	fill up to 45 mL
Adjust with suitably diluted NaOH/ H_2SO_4	pH 7.4 ± 0.1
Deionized water	fill up to 48 mL
Weight before autoclaving:	
Weight after autoclaving:	
Amount of water to be refilled:	

Glucose solution

Component	Weight [g]
Glucose · H_2O	12.22
Deionized water	fill up to 100 mL
Weight before autoclaving:	
Weight after autoclaving:	
Amount of water to be refilled:	

Thiamin solution

Component	Weight [g]
Thiamin · HCl	0.02
Deionized water	fill up to 20 mL
Sterile filtration into pre-sterilized vessel	

Magnesium solution

Component	Weight [g]
$MgSO_4 \cdot 7\ H_2O$	1.00
Deionized water	fill up to 20 mL
Sterile filtration into pre-sterilized vessel	

Trace elements solution

Components	Weight [g]
$ZnSO_4 \cdot 7\ H_2O$	0.011
$CuSO_4 \cdot 5\ H_2O$	0.010
$MnSO_4 \cdot H_2O$	0.006
$FeCl_3 \cdot 6\ H_2O$	0.835
Titriplex III	0.668
$CoCl_2 \cdot 6\ H_2O$	0.011
$CaCl_2 \cdot 2\ H_2O$	0.040
Deionized water	fill up to 20 ml
Sterile filtration into pre-sterilized vessel	

Final mix of stock solutions:

Components	Volume [mL]
Polysaccharide solution	30
Salt mix	48
Glucose solution	0.9
Magnesium solution	1.0
Vitamine solution	1.0
Trace elements solution	0.1
Deionized water	19
Enzyme mix: add not until starting an experiment, please see Technical Sheet	
